# Supplementary material for: Quartile coefficient of variation is more robust than CV for traits calculated as a ratio
Source: Sci Rep. 2023 Mar 22;13:4671. doi: 10.1038/s41598-023-31711-8 (PMC10033673; doi:10.1038/s41598-023-31711-8)
Supplement: Supplementary file 1 — Supplementary Information. [file 41598_2023_31711_MOESM1_ESM.docx]

**Appendix A**

**Taylor approximation of mean and standard deviation of ratios**

Consider random variables *x* and *y*, where y has support [0,∞]. Mean and standard deviation of $x/y=f\left( x,y \right)$ can be approximated by Taylor series expansion.

The first order Taylor expansion of $f\left( x,y \right)$ around $\left( \theta_{x},\theta_{y} \right)$ is

$$f\left( x,y \right)\approx f\left( \theta_{x},\theta_{y} \right)+\left( x-\theta_{x} \right)\frac{\partial f}{\partial x}\left( \theta_{x},\theta_{y} \right)+\left( y-\theta_{y} \right)\frac{\partial f}{\partial y}\left( \theta_{x},\theta_{y} \right)$$

Better precision can be reached by using second-order expansion:

$$f\left( x,y \right)\approx f\left( \theta_{x},\theta_{y} \right)+\left( x-\theta_{x} \right)\frac{\partial f}{\partial x}\left( \theta_{x},\theta_{y} \right)+\left( y-\theta_{y} \right)\frac{\partial f}{\partial y}\left( \theta_{x},\theta_{y} \right)+\frac{1}{2}\left\{ \left( x-\theta_{x} \right)^{2}\frac{\partial^{2}f}{\partial x^{2}}\left( \theta_{x},\theta_{y} \right)+\left( y-\theta_{y} \right)^{2}\frac{\partial^{2}f}{\partial y^{2}}\left( \theta_{x},\theta_{y} \right)+2\left( x-\theta_{x} \right)\left( y-\theta_{y} \right)\frac{\partial^{2}f}{\partial x\partial y}\left( \theta_{x},\theta_{y} \right) \right\}$$

Let the finite mean of x and y be $\mu_{x}=E\left( x \right)$ and $\mu_{y}=E\left( y \right)$. Let us choose the expansion point to be $\left( \mu_{x},\mu_{y} \right)$. Using the property of mean that when *a* and *b* are constant, $E\left( aX+bY \right)=aE\left( X \right)+bE\left( Y \right)$, the first order approximation of $E\left( f\left( x,y \right) \right)$ is

$$E\left( f\left( x,y \right) \right)\approx f\left( \mu_{x},\mu_{y} \right)+E\left( x-\mu_{x} \right)\frac{\partial f}{\partial x}\left( \mu_{x},\mu_{y} \right)+E\left( y-\mu_{y} \right)\frac{\partial f}{\partial y}\left( \mu_{x},\mu_{y} \right)$$

Since $E\left( x-\mu_{x} \right)=0$ and $E\left( y-\mu_{y} \right)=0$

$$E\left( \frac{x}{y} \right)=E\left( f\left( x,y \right) \right)\approx f\left( \mu_{x},\mu_{y} \right)=\frac{\mu_{x}}{\mu_{y}}$$

We can get a more precise result by using a second-order approximation with the same expansion point:

$$f\left( x,y \right)\approx f\left( \theta_{x},\theta_{y} \right)+\frac{1}{2}\left\{ \sigma_{x}^{2}\frac{\partial^{2}f}{\partial x^{2}}\left( \mu_{x},\mu_{y} \right)+\sigma_{y}^{2}\frac{\partial^{2}f}{\partial y^{2}}\left( \mu_{x},\mu_{y} \right)+2\mathrm{Cov}\left( x,y \right)\frac{\partial^{2}f}{\partial x\partial y}\left( \mu_{x},\mu_{y} \right) \right\}$$

where $\sigma_{x}^{2}=E\left( \left( x-\mu_{x} \right)^{2} \right)$ and $\sigma_{y}^{2}=E\left( \left( y-\mu_{y} \right)^{2} \right)$ are variances of x and y, respectively, while $\mathrm{Cov}\left( x,y \right)=E\left( \left( x-\mu_{x} \right)\left( y-\mu_{y} \right) \right)$ is their covariance.

If $f\left( x,y \right)=x/y$ then

$$\frac{\partial^{2}f}{\partial x^{2}}\left( x,y \right)=0$$

$$\frac{\partial^{2}f}{\partial y^{2}}\left( x,y \right)=\frac{2x}{y^{3}}$$

$$\frac{\partial^{2}f}{\partial x\partial y}\left( x,y \right)=-\frac{1}{y^{2}}$$

Therefore,

$$E\left( \frac{x}{y} \right)\approx\frac{\mu_{x}}{\mu_{y}}-\frac{cov\left( x,y \right)}{\mu_{y}^{2}}+\frac{\sigma_{y}\mu_{x}}{\mu_{y}^{3}}$$

For approximating the standard deviation of the ratio we can use that

$$D\left( f\left( x,y \right) \right)=\sqrt{E\left( \left( f\left( x,y \right)-E\left( f\left( x,y \right) \right) \right)^{2} \right)}$$

Using that $E\left( f\left( x,y \right) \right)\approx f\left( \mu_{x},\mu_{y} \right)$

$$D\left( f\left( x,y \right) \right)\approx\sqrt{E\left( \left( f\left( x,y \right)-f\left( \mu_{x},\mu_{y} \right) \right)^{2} \right)}$$

Then using the first order Taylor expansion around $\left( \mu_{x},\mu_{y} \right)$

$$D\left( f\left( x,y \right) \right)\approx\sqrt{E\left( \left( f\left( \mu_{x},\mu_{y} \right)+\left( x-\mu_{x} \right)\frac{\partial f}{\partial x}\left( \mu_{x},\mu_{y} \right)+\left( y-\mu_{y} \right)\frac{\partial f}{\partial y}\left( \mu_{x},\mu_{y} \right)-f\left( \mu_{x},\mu_{y} \right) \right)^{2} \right)}=\sqrt{E\left( \left( \left( x-\mu_{x} \right)\frac{\partial f}{\partial x}\left( \mu_{x},\mu_{y} \right)+\left( y-\mu_{y} \right)\frac{\partial f}{\partial y}\left( \mu_{x},\mu_{y} \right) \right)^{2} \right)}=\sqrt{E\left( \left( \left( x-\mu_{x} \right)\frac{\partial f}{\partial x}\left( \mu_{x},\mu_{y} \right) \right)^{2}+\left( \left( y-\mu_{y} \right)\frac{\partial f}{\partial y}\left( \mu_{x},\mu_{y} \right) \right)^{2}+\left( x-\mu_{x} \right)\frac{\partial f}{\partial x}\left( \mu_{x},\mu_{y} \right)\left( y-\mu_{y} \right)\frac{\partial f}{\partial y}\left( \mu_{x},\mu_{y} \right) \right)}=\sqrt{\sigma_{x}^{2}\left( \frac{\partial f}{\partial x}\left( \mu_{x},\mu_{y} \right) \right)^{2}+\sigma_{y}^{2}\left( \frac{\partial f}{\partial y}\left( \mu_{x},\mu_{y} \right) \right)^{2}+\mathrm{Cov}(x,y)\frac{\partial f}{\partial x}\left( \mu_{x},\mu_{y} \right)\frac{\partial f}{\partial y}\left( \mu_{x},\mu_{y} \right)}$$

If $f\left( x,y \right)=x/y$ then

$$\frac{\partial f}{\partial x}\left( x,y \right)=\frac{1}{y}$$

$$\frac{\partial f}{\partial y}\left( x,y \right)=-\frac{x}{y^{2}}$$

Therefore

$$D\left( \frac{x}{y} \right)\approx\sqrt{\frac{\sigma_{x}^{2}}{\mu_{y}^{2}}-\frac{\sigma_{y}^{2}\mu_{x}^{2}}{\mu_{y}^{4}}-\frac{\mathrm{Cov}\left( x,y \right)\mu_{x}}{\mu_{y}^{3}}}=\sqrt{\frac{\mu_{x}^{2}}{\mu_{y}^{2}}\left( \frac{\sigma_{x}^{2}}{\mu_{x}^{2}}-\frac{\sigma_{y}^{2}}{\mu_{y}^{2}}-\frac{\mathrm{Cov}\left( x,y \right)}{\mu_{x}\mu_{y}} \right)}=\frac{\mu_{x}}{\mu_{y}}\sqrt{\frac{\sigma_{x}^{2}}{\mu_{x}^{2}}-\frac{\sigma_{y}^{2}}{\mu_{y}^{2}}-\frac{\mathrm{Cov}\left( x,y \right)}{\mu_{x}\mu_{y}}}$$

**Appendix B**

**R function for calculating four alternative indices of relative variation**

cv<-function(x, method=c("standard","lognorm","geometric","quartile"))

{

method<-match.arg(method)

if (method=="standard") return(sd(x)/mean(x))

if (method=="lognorm") return(sqrt(exp(var(log(x)))-1))

if (method=="geometric") return(exp(sd(log(x)))-1)

if (method=="quartile") return(IQR(x)/sum(quantile(x,probs=c(0.25,0.75))))

}
